# The people’s game: evolutionary perspectives on the behavioural neuroscience of football fandom

**DOI:** 10.3389/fpsyg.2024.1517295

**Published:** 2025-01-10

**Authors:** Matt Butler, Gurjot Brar, Riadh Abed, Henry O’Connell

**Affiliations:** ^1^Neuropsychiatry Research and Education Group, King’s College London, London, United Kingdom; ^2^Trinity College Dublin, Limerick, Ireland; ^3^University of Limerick, Limerick, Ireland; ^4^Retired Consultant Psychiatrist, London, United Kingdom; ^5^Health Service Executive, Portlaoise, Ireland; ^6^School of Medicine, University of Limerick, Limerick, Ireland

**Keywords:** evolution, psychiatry, football, soccer, sports fan behaviour

## Abstract

Association football (soccer) is the world’s most popular sport. Transculturally, fans invest significant resources following their teams, suggesting underlying psychological universals with evolutionary origins. Although evolutionary science can help illuminate the ultimate causes of human behaviour, there have been limited modern evolutionary perspectives on football fandom. In this paper, we consider evolutionary perspectives on football fandom from a behavioural neuroscientific standpoint. We discuss how the appeal of football may arise through the low-scoring and highly variable outcomes of games; we relate this to the neuroscience of reward prediction errors and motivation. We highlight recent research on the psychobiological responses to ritual, including endorphin release, which may reduce anxiety and facilitate group bonding. We discuss the prosocial and anxiety-sublimating effects of the matchday ritual and argue that football may be a special case whereby ritual behaviour does have a small effect on the outcome of interest. We discuss the psychology of ingroup and outgroup effects of fandom and argue that, although resource scarcity can sometimes lead to aggression, that larger inter-group effects can be positive. We comment on the socioemotional developmental aspects of football fandom, and note how group identification may lead to displays of sacrifice. We finish with a discussion of whether, in the era of social prescribing, football could be seen as a psychiatrist’s tool. We conclude with suggestions on how the positive aspects of football can be emphasised through evolutionary perspectives, and how future research on football fandom may inform evolutionary understanding of humans *writ large*.

## Introduction

The roots of football and football fans run deeply. The oldest archaeological evidence of spectator sports is the 8th Century BC stadium at Olympia (Guttmann, 1981). Proto-football was played across cultures throughout history, including between whole towns during religious holidays (Goldblatt, 2007). In the late 19th century, association football (soccer) was codified, and football fans emerged (Walvin, 1975).

Football rapidly spread across the globe. It is now the world’s most popular sport, with an estimated 3.5 billion fans (World Population Review, 2024). The yearly revenue of European football alone amounts to around €35 billion (Deloitte Sports Business Group, 2024). Historically, football has been most watched by men, however the recent explosion in the women’s game is rebalancing this gender bias (Leslie-Walker and Mulvenna, 2022). Over recent years, the landscape of football fandom has shifted from that of a purely physical, local experience (i.e., watching in stadia) to a scalable, digitalised, television and media-driven experience. This changing landscape is altering the ways that many fans engage with the game (Woods and Ludvigsen, 2022).

Those who follow football teams often do so with significant attachment and commitment, sometimes to the bewilderment of those outside of the game (Newson et al., 2023), The apparent universality of fandom suggests that there are underlying psychological and behavioural universals. Evolutionary perspectives can help understand both the proximate (“how”) mechanisms underpinning such human behaviour, as well as ultimate (“why”) explanations (Nesse, 2019).

Humans are an ultra-social species uniquely and intensely dependent on culture (Henrich, 2015). Football is an example of a cultural innovation that has resonated with deep-seated, evolved, species-specific psychological and emotional systems (Morris, 1981). Nevertheless, to our knowledge, there have been no recent syntheses of evolutionary perspectives on football from a broad behavioural neuroscientific standpoint.

In this paper, we apply evolutionary principles to the study of a particular form of human social behaviour which is practiced in transcultural settings. We aim to illustrate how seemingly “irrational” behaviours of football fans can be understood, the deep evolutionary origins for such behaviours, and their potential health and social benefits.

We focus on four principal topics: the sporadic “reward” of goals and resultant effects on motivation, the ritualistic aspects of attending matches, the social dimensions of fandom, and the effects of football spectatorship on socio-emotional development. We link these discussions to the current evidence on football in mental health disorders.

### Reward and motivation in football fans

We begin by suggesting some non-exhaustive evolutionary reasons for why people might be motivated to enjoy sport. Firstly, sportspeople display physical attributes which may correlate with the ability to obtain resources (e.g., stamina, strength, speed) (Apostolou and Athanasiou, 2016). Secondly, sportspeople display skill, prowess, and expertise; potentially salient phenomena to an animal adapted to cultural learning (Mumford, 2013). Thirdly, competition is salient for humans, whose evolution was driven in part by periods of resource scarcity (Apostolou and Athanasiou, 2016). Fourthly, humans are a culture-dependent narrative species (Gottschall, 2012), and sport often produces compelling and unpredictable narratives with satisfying conclusions (Gleaves, 2017).

**Figure 1:**
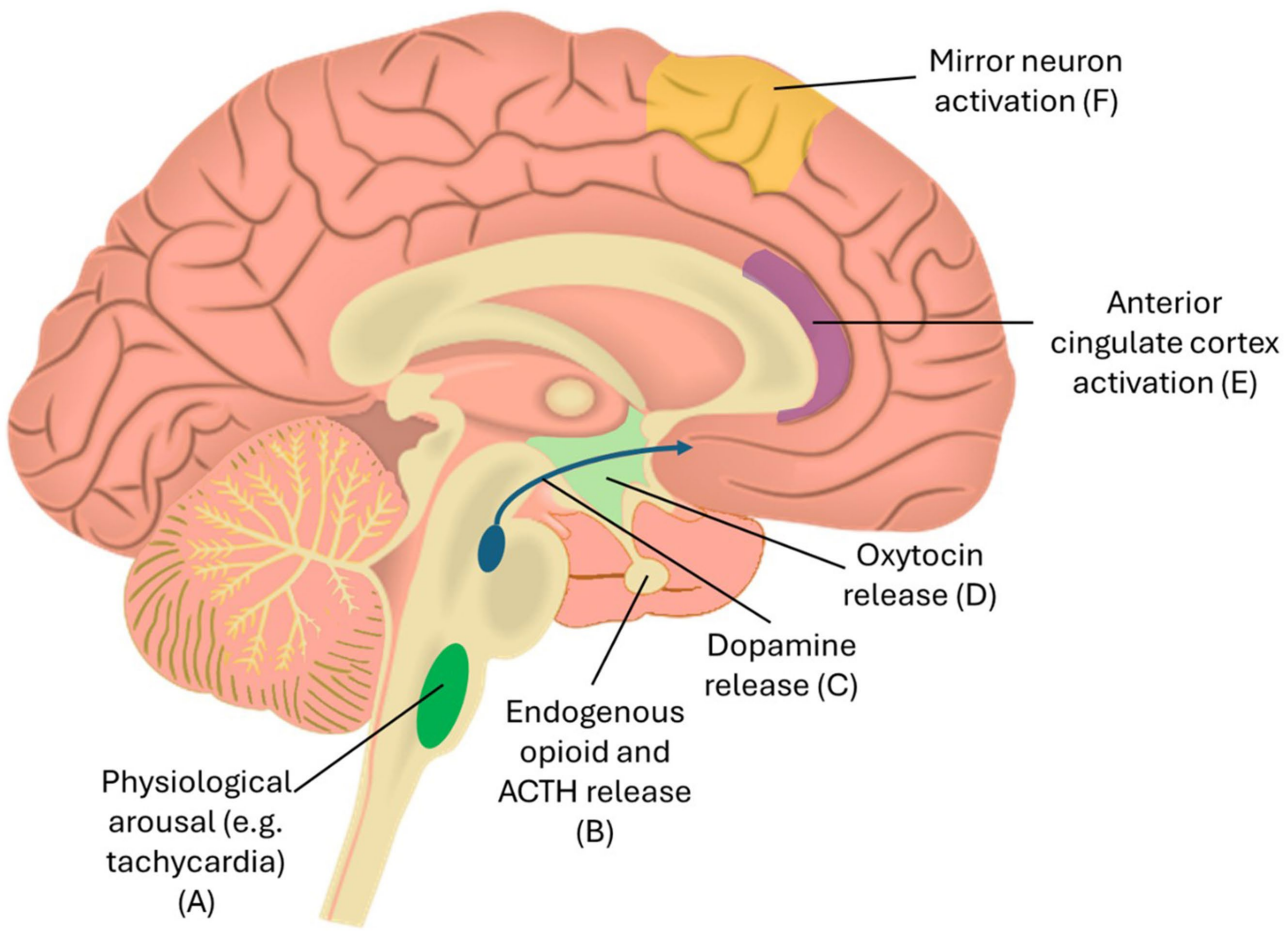
Your brain on football. **(A)** The experience of watching the game leads to physiological arousal, for example tachycardia. **(B)** The pituitary gland may release adrenocorticotropic hormone (ACTH) leading to cortisol production in the adrenal glands; endogenous opioids may also be released by the pituitary in response to the ritual aspects of the matchday experience. **(C)** Dopamine release in the mesolimbic pathway may be associated with the salience of expectation and reward (i.e., goals) (see Figure 2). **(D)** Oxytocin may be released by the hypothalamus in response to group identification and may be implicated in strengthening within group bonds. **(E)** Parts of the limbic system such as the anterior cingulate cortex are activated by watching goals. **(F)** The embodied feeling of fandom may arise in part due to the activation of mirror neurons in the motor cortex and related areas whilst watching a game.

But why do some football fans become obsessed with this particular game? One evolutionary suggestion is that football players communicate the greatest breadth of adaptive attributes (team spirit, aggression, speed, dexterity, stamina, and strength) in comparison to other sports (Apostolou and Athanasiou, 2016). It may also be because football is typically a low scoring game. This has at least two principal effects:

- Firstly, because goals are so rare, they are particularly euphoric when they occur. Goals likely stimulate the same dopaminergic pathways involved in, for example, winning random monetary prizes (Häusler et al., 2015); indeed, neuroimaging research has shown activation of limbic regions in fans who watch their team score (McLean et al., 2009; Duarte et al., 2017; Bilgiç et al., 2020) (Figure 1).
- Secondly, there is large natural variance in scorelines. Results are unpredictable; upsets can, and do, happen. Research on operant conditioning has indicated that reward-seeking behaviour is particularly pronounced when the rewards themselves are unpredictable and occur at random intervals (Pritchett and Mulder, 2004).

Although commonly understood as a “pleasure” neurotransmitter, dopamine release, particularly in the mesolimbic pathway, is also implicated in the processing of reward prediction errors (i.e., when outcomes of interest are better than expected) (Diederen and Fletcher, 2021). This dopaminergic signalling can lead to animals seeking to repeat behaviours associated with unpredicted rewarding outcomes (Keiflin and Janak, 2015).

Indeed, casinos and bookmakers try their best to hijack this innate phenomenon to compel users to repeat spending; games that reward at unpredictable intervals, such as electronic gaming machines, are significantly associated with addictive behaviours (Zack et al., 2020; Anselme, 2013). It has been suggested that failures of updating prediction errors might be implicated in the propensity to develop for example, gambling addiction (Keiflin and Janak, 2015).

Although the unpredictability of goals may produce repeated reward-seeking behaviour (i.e., a desire to repeatedly watch more matches), there is little to suggest that watching football can become a behavioural addiction, in even the most obsessive of fans (Kardefelt-Winther et al., 2017). Nevertheless, some fans are at risk of other behavioural disorders, such as gambling addictions in part due to the saturation of the game with gambling advertisements (Jones et al., 2020).

### Going to the match: football as a ritual

#### Physical effects of fandom

Watching a football match is a physical act. On matchdays, significant variation in physiological markers (such as raised heart rate) are observed (Elder et al., 1991). Testosterone (regardless of gender) (Burk et al., 2019), and cortisol is released; the extent of secretion may correlate with devotion to the team (Newson et al., 2020) (see Figure 1). Matches are associated with adverse cardiovascular events (thankfully still rarely), particularly when a team loses (Wang et al., 2020).

Spectatorship is an embodied experience in which meaning is produced through empathetic immersion with the players. Think of the crowds’ reaction when a striker leaps up to head a perfectly placed cross: everyone synchronously nods their head in sympathetic encouragement. Indeed, neuroimaging studies have shown that areas of the motor cortex are activated by watching sports (Beilock et al., 2008). This may be due to mirror neurons, those which activate whilst witnessing others perform tasks. Mirror neurones have evolved in relation to culturally-transmitted skill learning, as well as the foundation of empathy via the mapping of other-related information onto self-related brain networks (Bonini et al., 2022).

#### The matchday as ritual

Being part of a matchday crowd can be described as a form of ritual (Xygalatas, 2022). By one understanding, rituals are the performance of a series of actions or gestures which have an inherent, culturally-determined meaning, but do not produce an appreciable effect on the external world (Graf, 2012). Rituals are likely to have arisen early in, or prior to, the evolution of *Homo sapiens* and are a human universal (McNamara, 2022), suggesting that they have had adaptive significance (Rossano, 2015).

Evidence for ritual behaviour appears in our evolutionary past: the peoples inhabiting Britain in the neolithic period trekked for hundreds of miles for winter feasts at Stonehenge (Madgwick et al., 2019). Sacred locality was seemingly as important to our ancestors as it is to football fans today, where the sense of place (i.e., the stadium) is indelibly linked to the sometimes profoundly emotional memories of matches (Bairner, 2014).

Rituals can unify and strengthen group bonds as well as exciting individuals in an ecstatic togetherness; Durkheim proposed the term “collective effervescence” to describe this phenomenon (Durkheim, 1912). Part of the experience of watching football in a stadium is behavioural synchrony (e.g., chanting, cheering, and jeering in unison) which arises within a highly charged atmosphere. For example, singing and chanting (often to the beat of a drum) is commonplace in football stadiums. Chanting can be one way of affirming identity to the club (Clark, 2006). The evolutionary history of singing (to a drumbeat) likely has ancient origins, perhaps even arising before the development of language (Rafferty et al., 2023).

#### Identity fusion

Research has suggested that the psychophysiological arousal experienced in such collective settings is associated with the release of endogenous opioids (Charles et al., 2020). Synchrony may act as a direct means of enhancing group cohesion via the release of such neurohormones that influence social bonding (Tarr et al., 2017). This can explain its recurrence throughout diverse human cultures and contexts (e.g., dance, prayer, marching, music-making, and sport) which provide an alternative to social bonding mechanisms such as grooming, which are prevalent in other great apes (Launay et al., 2016).

The psychophysiological response to ritual also promotes “identity fusion,” whereby individuals’ person and social identities merge (Páez et al., 2015; Lang et al., 2017). In football, a fan becomes identified with the (largely anonymous) crowd of fellow supporters in the stadium and beyond (Newson et al., 2023). The match itself is necessary, in that it scaffolds the experience (just as a religious ritual is necessary to bind the congregation), but it is not in itself sufficient for the formation of fanaticism, which requires the presence of a crowd (Dunbar, 2022).

#### Rituals as means to resolve uncertainty

Simple ritualised behaviours can serve to reduce anxiety (Lang et al., 2015). This is best illustrated by obsessive-compulsive disorder (OCD), in which an affected individual may feel a compulsion to perform repeated ritualised behaviours to resolve an obsession or anxious thought (Moulding and Kyrios, 2006). Provoking anxiety in healthy individuals can also spontaneously induce ritualistic behaviour; as with OCD, uncomfortable feelings of uncertainty may motivate individuals to seek order through the performance of a familiar, repetitive act (Lang et al., 2015).

Larger social rituals may arise or become more prominent in challenging periods, such as in times of resource stress. As an example, rainmaking rituals are practiced across cultures, particularly in times of drought (Murray and Xing, 2020). Rituals may help to convince a group that they are exerting control over a situation, which serves to resolve uncomfortable uncertainty. Rituals may therefore improve psychological wellbeing, and are employed in transcultural settings as a healing process, particularly when there is limited access to mainstream psychiatric services (Sax, 2014; Xygalatas et al., 2019).

Matchday crowds perform ritualistic acts which may serve to help resolve anxiety around the unpredictability of the result. Furthermore, football may represent an unusual case whereby the ritual *does make a small but significant difference* (Pollard, 2006a). Research on the “home advantage” phenomenon (most teams perform better at home, where there are more of their own supporters), have suggested that partisan crowds do lead to increased goals for home teams and increased yellow cards for away teams (Pollard, 2006b). Systematic analysis of the crowd-less “ghost games” played without spectators in the COVID-19 pandemic suggests that lack of a crowd decreases this home advantage (Leitner et al., 2023).

Football matches may therefore induce all the psychophysical benefits of ritual, but may be even more satisfying because the ritual behaviour does increase the chances of their team winning. The responsibility for this is shared across the crowd of fans, so as not to be burdensome to any one individual. In the era of social prescribing, the putative psychophysical benefits of social ritual warrants further research (Fixsen and Polley, 2020).

### Social dimensions of fandom

#### Ingroups and outgroups

Humans are tribalistic social animals who spontaneously form groups with complex, bonded relationships. The Social Brain Hypothesis proposes that the human neocortex evolved and enlarged in part to cope with the cognitive demands of such intricate relationships (Dunbar, 2016). Evolutionary pressures likely selected for the sharing of knowledge and experience, as well as the pooling of resources, which is adaptive at the group level (Henrich, 2015).

Humans can become biased towards ingroups and antagonistic towards outgroups, classically illustrated by the 1954 Robber’s Cave experiment, and since replicated in various forms (Berkowitz and Sherif, 1967). In football, this tendency leads to the partisan support of one’s own team (the ingroup) which is viewed favourably in comparison to rival teams (the outgroups).

**Figure 2:**
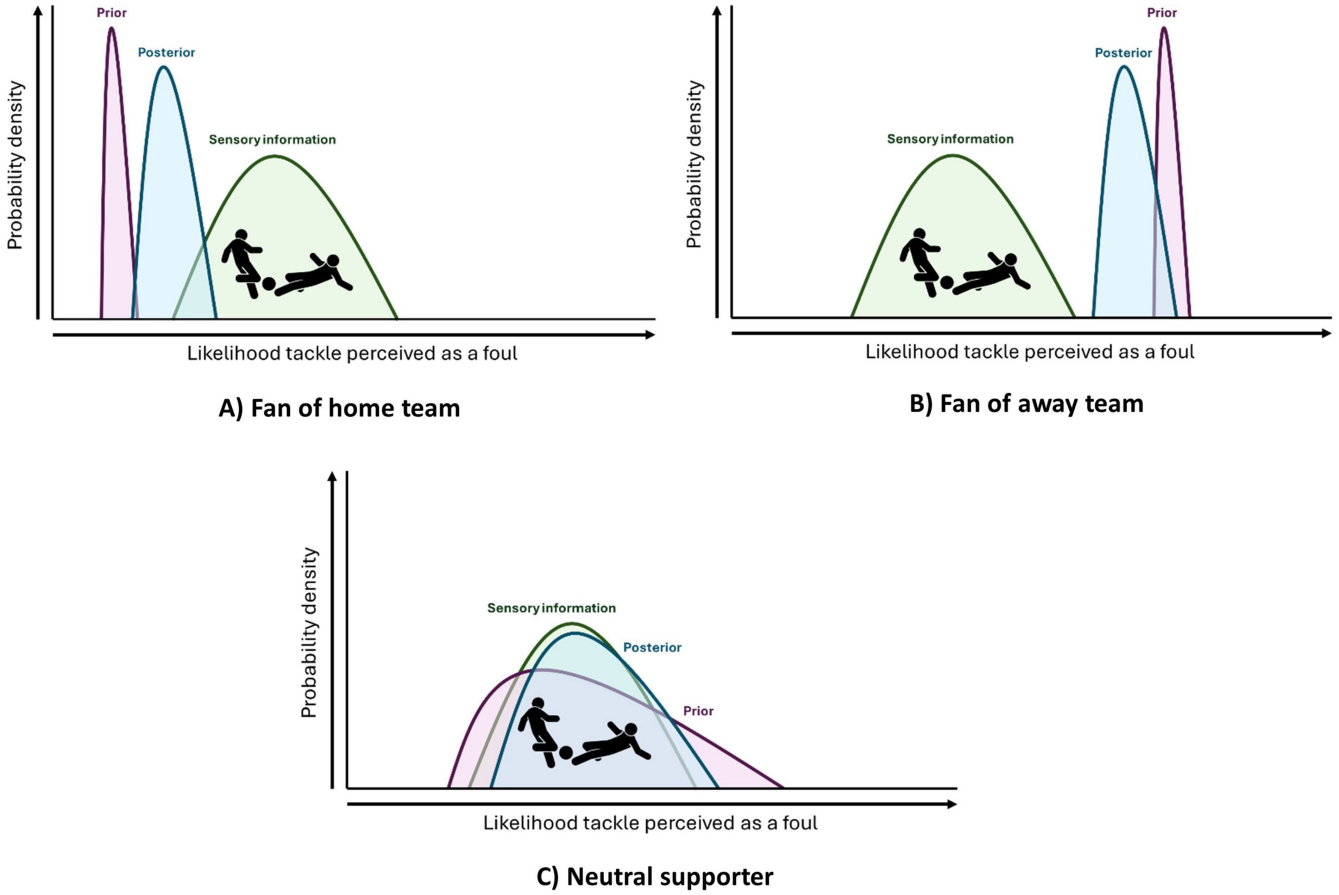
Predictive processing as applied to a football tackle. In this example, a home player has tackled an away player in a game between two fierce rivals. The tackle is watched by a partisan home fan **(A)**, a partisan away fan **(B)** and a neutral supporter **(C)**. The prior probability of the tackle being a foul (in purple) is influenced by fan group status. In the case of the home team fan, there is a precisely held prior that their player tackles fairly, and therefore the experience of the tackle (the posterior, blue) is less influenced by the ambiguous sensory input; hence, the tackle is perceived as fair. In the case of the away fan, the precise prior is on the opposite side of the probability scale, and the tackle is perceived as a foul. In the case of the neutral supporter, the prior is held with less precision, and hence the experience of the ambiguous tackle (posterior) is weighted more heavily towards the sensory input.

Humans may develop biased perceptions which favour members of ingroups, at the preconscious as well as conscious level (Molenberghs et al., 2013) (Figure 2). Understood via the Bayesian or predictive processing model of perception, ingroup status may alter neuronal “priors,” which biases perception towards the ingroup (Moradi et al., 2015). This enables individuals to heuristically predict, rather than simply respond to, their social environment (Pezzulo et al., 2022; Seth, 2021). Heuristic predictions may be adaptive, as they require less cognitive effort in comparison to considering all available information (Gigerenzer and Gaissmaier, 2011).

Football fans are notoriously biassed towards their own teams (Na et al., 2019), as evidenced by perceptions of the number of fouls committed (Hastorf and Cantril, 1954), and overall contribution of their team to a match (Huff et al., 2017). Being a fan may increase the strength and precision of prior beliefs about the team (e.g., our players tackle fairly); correspondingly, sensory evidence (e.g., visual input of a tackle) is given less relative weight in the resultant Bayesian modelling, which leads to “posteriors” (conscious experience of the tackle) not being updated to the same degree as they would be in an unbiassed spectator. For worked examples of this phenomenon (please see Figure 2).

#### The narcissism of small differences

The strongest rivalries between fanbases tends to happen in the context of crowded resource niches. This may be competition for support (e.g., local derbies), or for trophies. Sigmund Freud’s concept of the “narcissism of small differences”—the more a relationship or community shares commonalities, the more likely the people in it are to engage in interpersonal feuds and mutual ridicule—seems a particularly apt description (Freud, 1930).

Fans of rival clubs have a particular tendency to experience *schadenfreude* – pleasure at others’ misfortune. This is a complicated emotion with a social dimension. Schadenfreude has been shown, including in studies of football fans, to be associated with the strengthening of ingroup identification, particularly in times of hardship (i.e., following losses), suggesting an adaptive social role for the emotion (Hoogland et al., 2015; Leach and Spears, 2009). The degree to which schadenfreude should be tolerated or encouraged is a question with complex ethical ramifications.

All ingroups, including communities, gatherings, and professions (as well as football fans), develop a specific vocabulary and means of referring to objects or concepts which can serve as shorthand, but can also signal ingroup membership and obfuscate concepts from outsiders (Efferson et al., 1979). Slang, lingo, and shibboleths (such as player nicknames, “in-joke” chants, and particular fashion styles) may serve as signifiers which maintain distinctions. Overt displays of support, for example costly journeys to away matches, may have evolutionary origins as means of displaying devotion to a group via self-sacrifice (De Dreu et al., 2014).

Seen through an evolutionary lens, these ingroup markers may serve to encourage parochial altruism, and facilitate the detection of outgroup individuals and “freeloaders.” Humans are attuned to social behaviour within groups and can become frustrated when freeloaders attempt to extract benefits from a group they are perceived not to have contributed to (Avilés, 2002). There is some (albeit weak) evidence, including from research on football fans, that parochial altruism may be regulated by the hormone oxytocin (de et al., 2010; Luo et al., 2019; Schiller et al., 2020). Oxytocin administration has been suggested as a treatment for altered social functioning in numerous psychiatric disorders including autistic spectrum disorder, schizophrenia, borderline personality disorder, and social anxiety disorders (De Dreu and Kret, 2016).

#### Hooliganism is a problem, but football is not war

Since antiquity, sports have been compared to battle, with a particular emphasis on attributes such as aggression, strength, and ruthlessness (Lombardo, 2012). War, too, has been a common metaphor in football, partly due to its association with unsavoury incidents of crowd violence (Mangan, 2004).

Hooliganism at football matches remains an occasional issue. There are some suggestions that hooliganism may be associated in some cases with untreated neurodevelopmental and mental health disorders (Smith et al., 2024; van Ham et al., 2022). There is also a replicated body of data to suggest that some international tournament matches are associated with increased alcohol consumption and incidents of alcohol-related domestic violence (Trendl et al., 2021).

Nevertheless, the experience of most fans is not one of violence, and disorder is perpetrated by a minority. There are issues with stigmatising all football fans as inherently violent (File and Worlledge, 2023), and the evolutionary history of human intergroup competition suggests that cooperation is at least as important as aggression (evidence suggests this applies to football fans too) (Newson et al., 2023).

Indeed, there are emerging data to suggest that football can serve as a means to build social cohesion. Studies on football (playing, not watching) in the Middle East suggests that it can improve relationships and admixing between players of differing religions (Mousa, 2020). Mohamed Salah, a particularly gifted Muslim footballer who plays for Liverpool, provides another example. Salah often performs sujood (the Muslim act of devotional prostration) after scoring, and his signing for Liverpool was associated with a small reduction in hate crimes in Merseyside, and a halving of Islamophobic tweets from Liverpool fans (Alrababa’h et al., 2021).

#### Social (and antisocial) media

The explosion of social media has increased the opportunities for fans to engage with their team via the internet (a phenomenon sometimes called “digital fandom”). This undoubtedly increases connections between fans, and maximises exposure to media and opinion about football. But football discourse on social media is not without problems, and there have been repeated reports of abuse (including racist abuse) of players or other fans, perpetrated by online social media users behind a cloak of anonymity (Kilvington and Price, 2019).

Evolutionary theories around social media tend to emphasise the problems of environmental mismatch, whereby social media is designed to co-opt innate mechanisms of our socially-orientated brains, leading to repeated, and sometimes harmful, use. Transient rewards from “Likes” and online interactions belies an overwhelming social landscape which we are ill-adapted to. Harmful social media use has been associated with low mood, poor self-esteem, and behavioural addiction; for a detailed evolutionarily-informed discussion of social media use, please refer to Lim and Tan, 2024.

### Socialisation and the emotions of football

#### The scarf my father wore

Many football fans end up supporting the same football team as family members, particularly if they are taken to games as a child. It is self-evident that children have evolved an innate tendency to copy the behaviours, mannerisms, and preferences of their primary caregivers, and this often applies to football too.

Humans are unique in that emotional development continues far after birth, and is influenced by cultural learning (Savina and Wan, 2017; Michalska and Davis, 2019). Watching football matches with peers and adults may represent a rite-of-passage for some children, a means by which both collective and individual emotional development occurs, perhaps through the generation of emotionally-salient memories (Bairner, 2014) and the freedom to behave with less restriction on emotional and physical expression than in other areas of life (Pringle, 2004).

For older fans, this may result in bittersweet nostalgia, an emotion which saturates football. Nostalgia was first described in medical literature in the 17th Century (Anspach, 1934), however its ubiquity suggests a deeper evolutionary past. The solid, unchanging history of a football club can become one of the emblems by which a fan group reaffirms its identity in the face of the uncertain and unpredictable future. Nostalgia may have adaptive function through fostering optimism and the buffering of adverse experiences (Yang et al., 2023). Nostalgia has been utilised in new therapeutic paradigms, for example in reminiscence therapy for dementia, which can explicitly focus on sporting memories, including of football (Coll-Planas et al., 2017; Sass et al., 2021).

#### The emotional game

During a football match there is a constant building up and letting down of emotions, and expectations are frustrated at least as often than they are rewarded. Football, for many, often means defeat and disappointment. Despite this, football fans, as is the case with humans generally (Soper, 2021), have a particular tendency to show optimism bias (Massey et al., 2011), a rose-tinted view of the world which may have important evolutionary underpinnings for motivation in the face of adversity.

The idea that football fans “cut off reflected failure” by identifying with their club less following defeat was suggested in older literature (Snyder et al., 1986). Nevertheless, there is counter-evidence, suggesting fans are more likely to “sacrifice” themselves for the sake of other fans of their team (the ingroup) after a defeat (Newson et al., 2023; Whitehouse et al., 2017). Perhaps this could be named the “doubling down after a defeat” effect: the shared agony of losing games glues fans together as much as the collective ecstasy of wins (Newson et al., 2023; Whitehouse et al., 2017). This may have important evolutionary underpinnings, whereby strengthening bonds within a group is particularly important in times of hardship.

Nevertheless, very strong identity fusion with a football club may sometimes be maladaptive (‘negative over-identification’), and may have arguably too significant an effect on mood and wellbeing. Defeats, even if routine, may affect overall mental wellbeing, with higher identifying fans experiencing anger as well as sadness (Crisp et al., 2007). There is some (weak) evidence that clinically significant low mood can occur in fans after their team is relegated (Banyard and Shevlin, 2001).

### Should football be prescribed by psychiatrists?

Given the positive psychosocial effects of football, should psychiatrists be asked to consider “prescribing” football? Playing football has been shown to have a small effect on physical and mental health of people with schizophrenia (Battaglia et al., 2013), and there is a tradition of utilising football in mental institutions in some countries (Nolot et al., 2012). Some have suggested that the sense of inclusion, purpose, and peer identification engendered by football may support resilience in mental health (Heun and Pringle, 2018). Other potentially beneficial effects include exposure to the outdoors and engaging in vigorous physical exercise.

Nevertheless, consensus on the quantitative effects of football fandom on mental health remains unresolved. Sporting events are recognised to transiently increase societal wellbeing (Keyes et al., 2023; Ramchandani et al., 2022); there is some evidence suggesting that national sporting events, including football World Cups, may temporarily decrease suicide (Encrenaz et al., 2012; Andriessen and Krysinska, 2009), but this has not been consistently replicated (Pichler et al., 2023). Although qualitative analyses are generally supportive of football’s role in supporting the mental health of interested people (Pringle, 2004; Ramchandani et al., 2022; Friedrich and Mason, 2017), there is no consistent evidence that football has positive effects on mental illnesses (Heun and Pringle, 2018).

## Conclusion

As with all communal cultural activities, football fandom is an inherently social phenomenon deeply rooted in our evolutionary past. The appeal of football may arise through the low-scoring and highly variable outcomes of games. Psychobiological responses to ritual, including endorphin release, may facilitate group bonding in the stadium and beyond. Ingroup identification may lead to displays of sacrifice for their own team.

Taking evolutionary perspectives on football fandom can help further our understanding of wider human behaviours. Understanding how fans differentially align their support to club and national teams may help understand the role and formation of local and national identities. Further research on negative over-identification is required to help illustrate how innate human traits can become maladaptive at their extremes. Finally, understanding football fans’ behaviour may help inform how lager groups, such as nations and religious groups, interact with each other, particularly in times of scarcity, hardship, and resource competition.

## Data Availability

The original contributions presented in the study are included in the article/supplementary material, further inquiries can be directed to the corresponding author.
